# Targeting myeloid-derived suppressor cells to attenuate vasculogenic mimicry and synergistically enhance the anti-tumor effect of PD-1 inhibitor

**DOI:** 10.1016/j.isci.2021.103392

**Published:** 2021-11-01

**Authors:** Yinan Li, Kailiang Qiao, Xiaoyun Zhang, Haoyang Liu, Heng Zhang, Zhiyang Li, Yanrong Liu, Tao Sun

**Affiliations:** 1 State Key Laboratory of Medicinal Chemical Biology and College of Pharmacy, Nankai University, Tianjin 300350, China; 2 Tianjin Key Laboratory of Molecular Drug Research, Tianjin International Joint Academy of Biomedicine, Tianjin 300450, China; 3 Molecular Pathology Institute of Gastrointestinal Tumors, Affiliated Hospital of Jining Medical University, Jining Medical University, Jining 272013, Shandong, China

**Keywords:** Cancer, Genetics

## Abstract

Myeloid-derived suppressor cells (MDSCs) enhance the proliferation of endothelial cells to stimulate angiogenesis. However, many aggressive malignant tumors do not have endothelial cell-dependent blood vessels in the early stage and instead generate microcirculation by forming vasculogenic mimicry (VM). To date, the relationship between MDSCs and tumor cells remains the focus of ongoing studies. In this work, MDSCs were co-cultured with mouse melanoma cells and can enhance proliferation and VM formation of melanoma cells. For MDSCs targeting, doxycycline (DOX) was found to selectively suppress PMN-MDSCs but has no influence on T cells. In addition, DOX pretreatment substantially reduced the promoting ability of MDSCs for the VM formation of B16-F10 cells. DOX also inhibited tumor growth and enhanced the antitumor activity of PD-1 inhibitors in C57BL6 and BALB/c mice subcutaneously inoculated with B16-F10 and 4T1 cells, respectively. In conclusion, the combination of DOX and PD-1 inhibitor could be an anticancer strategy.

## Highlights

• MDSCs accumulated in the B16-F10 tumor-bearing mice
• MDSCs promote the formation of vasculogenic mimicry
• Doxycycline selectively suppressed PMN-MDSCs
• Doxycycline combined with PD-1 inhibitor significantly inhibited tumor growth

## Graphical abstract

**Figure:**
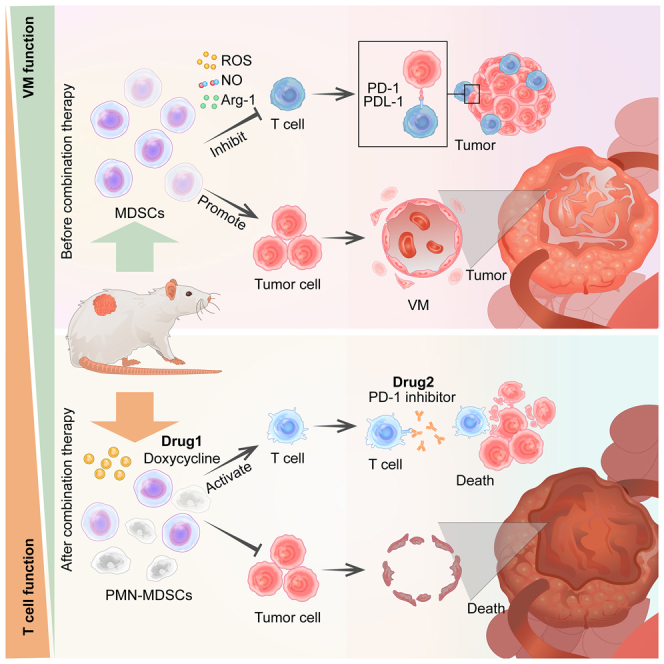

## Introduction

Tumor microenvironment is important in tumor development, including invasion and metastasis (Bissell and Radisky, 2001; Delgado-Bellido et al., 2017). Myeloid-derived suppressor cells (MDSCs) are one of its main driving forces (Umansky and Sevko, 2013), including polymorphonuclear MDSC (PMN-MDSC) labeled as CD11b^+^Ly6G^+^Ly6C^low^ and monocytic MDSC (M-MDSC) labeled as CD11b^+^Ly6G^−^Ly6C^high^ subpopulations. MDSCs targeting is a new cancer treatment strategy (Gabrilovich and Nagaraj, 2009; Ostrand-Rosenberg, 2010; Zou, 2006). They are a heterogeneous population of bone marrow-derived immature myeloid cells that are highly immunosuppressive and negatively regulate the immune responses during infection, cancer, and the maternal immune response to fetuses. These cells also promote tumor growth and metastasis by suppressing the anti-tumor immune response and controlling paracrine secretions that stimulate tumor cell proliferation, motility, and angiogenesis (Fukumura et al., 2006). MDSCs show a correlation with poor prognosis in human melanoma and with lymph node metastasis in patients with breast cancer (Montero et al., 2012; Poschke and Kiessling, 2012).

The PD1/PD-L1 axis plays an important role in immune escape. Blocking each of its components can promote the body's anti-tumor immune response and inhibit the proliferation and metastasis of tumor cells (Barber et al., 2006; Dong et al., 2004; Iwai et al., 2005; Strome et al., 2003). Although PD-1/PDL-1 inhibitors have good antitumor effects and have been approved by the US Food and Drug Administration for various tumor types, their efficacy is challenged by several factors (Chen and Han, 2015; Sheng et al., 2018), such as the tumor immunosuppressive microenvironment. MDSC is one of the important factors in the tumor immunosuppressive microenvironment. An increase in MDSCs count is related to PD-1 or PD-L1 inhibitor resistance (Li et al., 2021; Olivares-Hernández et al., 2021; Salewski et al., 2021; Shi et al., 2021; Uckun, 2021). Therefore, MDSC inhibitors combined with PD-1/PD-L1 inhibitors can achieve better antitumor effects than PD-1/PD-L1 inhibitors alone.

The anti-tumor activity of tetracyclines has been verified in many tumor types. Doxycycline (DOX) has been subjected to evaluation in clinical trials (Ali et al., 2017). It exerts multipotent drug activity by inhibiting angiogenesis, metastasis, ribosome activity, and mitochondrial function. However, its relationship with MDSCs has not been investigated. In this study, MDSCs promoted tumor cell proliferation and vasculogenic mimicry (VM) formation. DOX inhibited the proliferation of MDSCs and reduced the ability of VM formation. The treatment of DOX combined with PD-1 inhibitor exerted a stronger anti-tumor effect than treatment with single drugs.

## Results

### MDSCs increasingly accumulate in the bone marrow and spleens of B16-F10 tumor-bearing mice

**Figure 1:**
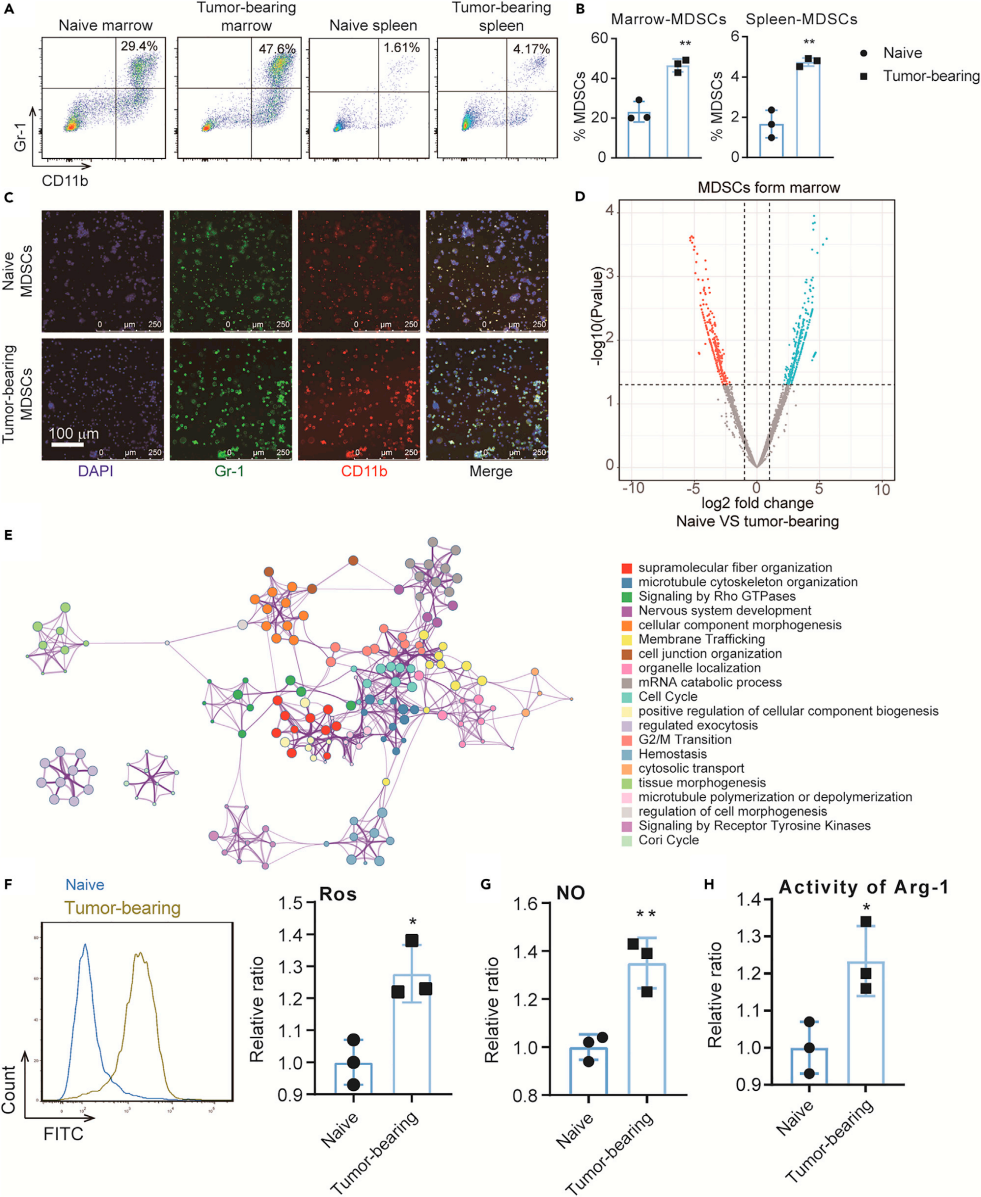
MDSCs accumulate in B16-F10 tumor-bearing mice (A) Representative dot plots of CD11b^+^Gr-1^+^ MDSCs in the bone marrows (left two panels) and spleen (right two panels) of naive mice or B16-F10 tumor-bearing mice. (B) Unpaired Student’s t test was done for statistical analyses of MDSC ratio. (C) MDSCs of bone marrow from naive and B16-F10 tumor-bearing mice labeled by CD11b (red), Gr1 (green), and nucleus (blue) to detect the expression of MDSC makers. (D) Volcano plot of the differentially expressed proteins of MDSCs between native mice and B16-F10 tumor-bearing mice (n = 3). (E) Enrichment analysis of differentially expressed proteins by Metascape (https://metascape.org). (F–H) Immunosuppressive function analysis of MDSCs including ROS level, NO production, and Arg1 activity. All statistical data are represented as mean ± SEM and Unpaired Student’s t test was done for statistical analyses. ∗p < 0.05, ∗∗p < 0.01.

After the tumor model was established, naive and B16-F10 tumor-bearing mice were sacrificed when their tumor volume reached approximately 1,000 mm^3^. Spleen and bone marrow cells were subsequently obtained. Flow cytometry was used to measure the proportion of MDSCs in the collected cell samples. We found that the MDSC proportion in B16-F10 tumor-bearing mice was significantly increased compared with that in the corresponding naive mice (Figures 1A and 1B). Immunofluorescence experiment confirmed the above results (Figure 1C). MDSCs from the bone marrow of naive and B16-F10 tumor-bearing mice were then sorted and analyzed by proteomics. A total of 955 differential proteins were identified. Enrichment analysis found that many pathways involving cell cycle and immune cell function were enriched (Figures 1D and 1E, Table S1). At the tumor site, MDSCs affect T cells to form an immunosuppressive microenvironment by producing Arg-1, iNOS, IDO, NOX2, and immunosuppressive cytokines. Therefore, the ROS level (Figure 1F), NO production (Figure 1G), and Arg-1 activity (Figure 1H) of MDSCs were measured. An increase in the above values was found in B16-F10 tumor-bearing mice compared with those in naive mice. Therefore, MDSCs accumulate in tumor-bearing mice and exert immunosuppressive effects.

### MDSCs promote the proliferation and VM formation of B16-F10 cells *in vitro*

**Figure 2:**
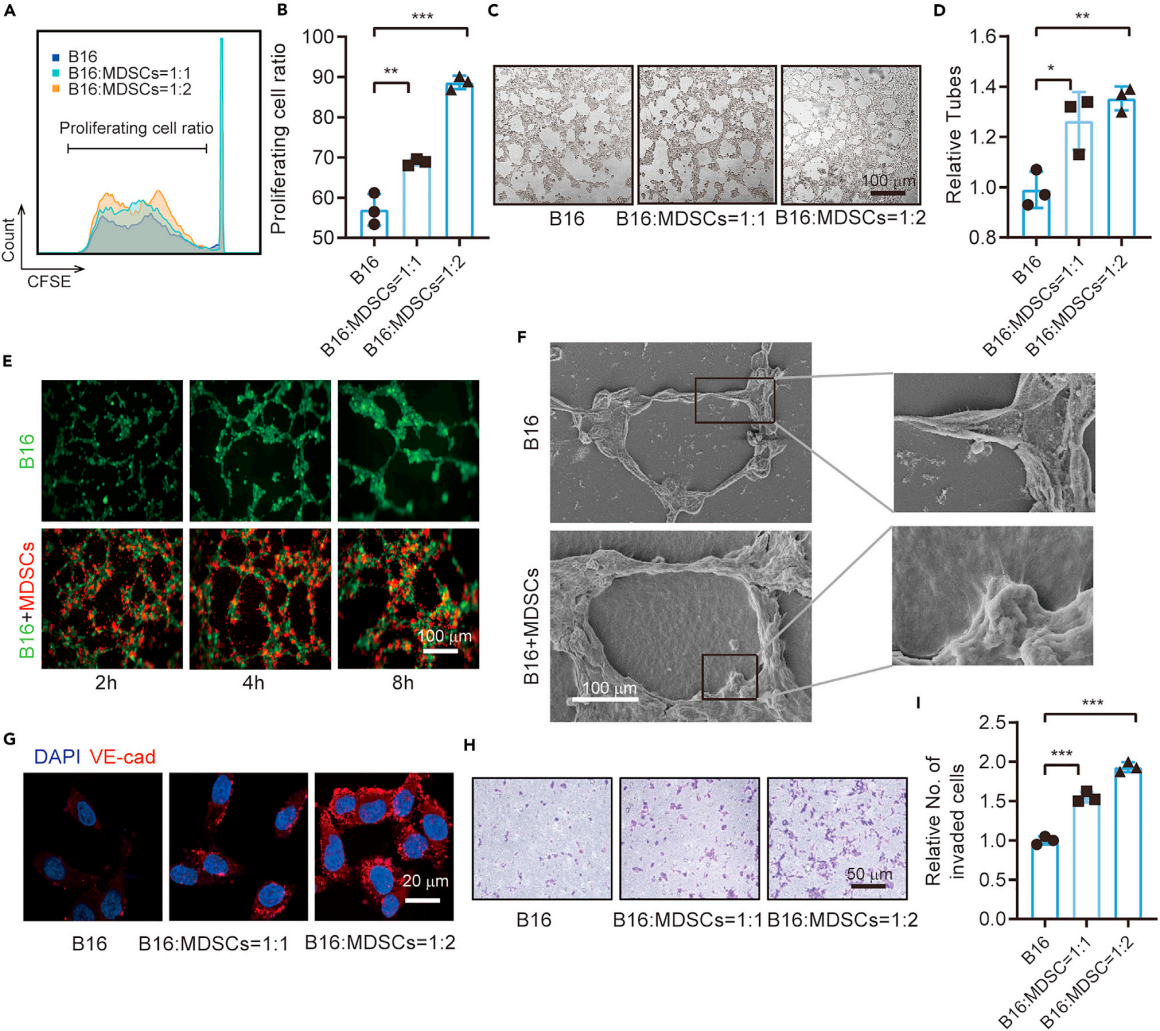
MDSCs promote proliferation and VM formation of B16-F10 cells *in vitro* (A) Representative CFSE histograms of co-cultured cells (B16-F10 and MDSCs) showing that the MDSCs from tumor-bearing mice dose dependently promoted the VM of B16-F10 cell line. (B) Statistical analysis of the ratio of proliferating cells. (C) Representative fields of tube formation of B16-F10 with or without MDSCs of different concentrations. (D) Statistical analysis of the relative tube number to evaluate the ability of VM formation for B16-F10 cells. (E) Living cell tracing for the co-culture of B16 cells labeled by GFP and MDSCs labeled by CM-DiI. (F) SEM image of VM showing that the co-culture of MDSCs and B16 promoted vascular mimicry and extracellular matrix secretion. (G) Immunofluorescence staining showing the expression of VM marker VE-cadherin. (H) Representative image of Transwell invasion assay. (I) Statistical analysis of the relative number of invaded cells. All statistical data are represented as mean ± SEM. Unpaired Student’s t test was done for statistical analyses. ∗p < 0.05, ∗∗p < 0.01, ∗∗∗p < 0.001.

MDSCs from the bone marrow in B16-F10 tumor-bearing mice were sorted for the co-culture experiment to verify the interaction between MDSCs and B16-F10 cells. CFSE staining revealed that the B16-F10 cells co-cultured with MDSCs (set B16-F10:MDSC ratios as 1:0, 1:1, and 1:2) had stronger proliferation ability than B16-F10 cells alone (Figures 2A and 2B). Furthermore, MDSCs promoted the VM formation of B16-F10 cells (Figures 2C and 2D). Living cell tracking results revealed that the MDSCs were usually located in the island of VM pattern formed by B16-F10 cells, and their accumulation on this island was dependent on the co-culture time (Figure 2E). SEM findings showed more extracellular matrix (ECM) secretions around the tube in the co-culture system than that in the B16-F10 cells alone (Figure 2F). The expression of the VM maker of B16-F10 cells, namely, VE-cadherin, was also increased in the co-culture system (Figure 2G). The Transwell invade assay was used to detect the malignant progression of B16-F10 cells and showed that MDSCs promoted the invading ability of B16-F10 cells (Figures 2H and 2I).

### DOX inhibits the promoting capability of MDSCs for the VM formation of B16-F10 cells

**Figure 3:**
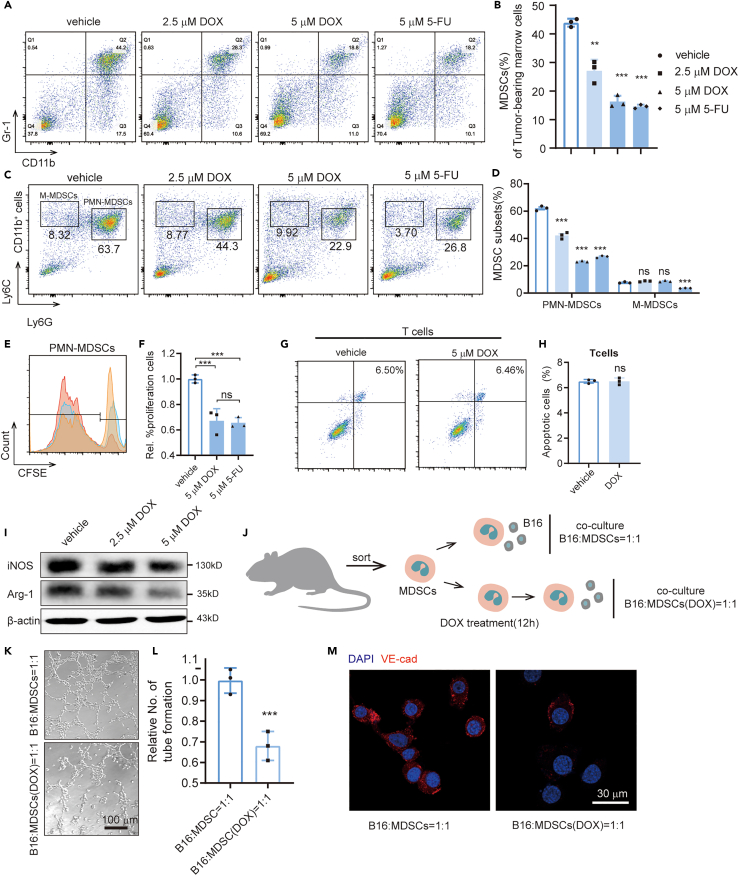
Doxycycline inhibits the promoting capability of MDSCs for VM formation *in vitro* (A) Flow cytometric analysis of the proportion of MDSCs in bone marrow from C57BL/6 tumor-bearing mice treated by different doses of doxycycline and 5-FU. (B) Statistical analysis of the ratio of MDSCs. (C) Flow cytometric analysis of the proportion of PMN-MDSCs (CD11b^+^Ly6G^+^Ly6C^low^) and M-MDSCs (CD11b^+^Ly6G^−^Ly6C^high^) in bone marrow from C57BL/6 tumor-bearing mice treated by different doses of doxycycline and 5-FU. (D) Statistical analysis of the ratio of MDSCs subsets. (E) Representative CFSE histograms of PMN-MDSCs. (F) Statistical analysis of the ratio of proliferating cells. (G) Representative dot plots for apoptosis of T cells after DOX treatment. (H) Statistical analysis of the apoptosis ratio of T cells. (I) Western blot to detect the levels of Arg-1 and iNOS in MDSCs after different doses of DOX treatment. (J) Schematic showing the co-culture of B16 and MDSCs with or without DOX treatment. (K–L) Representative images and statistical analysis of tube formation to evaluate the ability of co-cultured cells. (M) Immunofluorescence image to detect the expression of VE-cadherin of B16 cells. All statistical data are represented as mean ± SEM. Unpaired Student’s t test was done for statistical analyses. ∗∗p < 0.01, ∗∗∗p < 0.001.

DOX exerts anti-cancer activity and has been assessed as an anti-cancer agent in clinical trials. However, its mechanism remains unclear. The marrow cells of tumor-bearing mice were treated by DOX to determine whether this drug inhibits MDSCs. Meanwhile, 5-fluorouracil (5-FU), a drug that specifically inhibits MDSCs, was also evaluated. Flow cytometry showed that DOX reduced the proportion of MDSCs in the marrow cells *in vitro*, which has comparable effects with 5-FU (Figures 3A and 3B). To study which subtype of MDSCs that DOX can inhibits, we extracted bone marrow cells from B16-F10 tumor-bearing mice and labeled them with CD11b, Ly6C, and Ly6G to distinguish PMN-MDSCs and M-MDSCs. We found that DOX significantly inhibited the proportion of PMN-MDSCs but had no effect on M-MDSCs *in vitro*, whereas 5-FU has no selective inhibition for different subsets (Figures 3C and 3D). Then we evaluated the proliferation ability of PMN-MDSCs treated by DOX and 5-FU and found that both drugs inhibit the proliferation significantly. There is no significant difference for the inhibitory ability of the two drugs (Figures 3E and 3F). Previous study found that 5-FU can promote the apoptosis of MDSCs (Vincent et al., 2010). We also examined the effect of DOX on apoptosis in MDSCs and found that it is modest (Figure S1).

To further study whether DOX can influence the function of T cells, we programmed Annexin V FACS assay and found that DOX cannot promote the apoptosis of T cells (Figures 3G and 3H). Western blot was conducted to verify the influence of DOX on the immunosuppressive function of MDSCs. The levels of Arg-1 and iNOS were significantly decreased after DOX treatment (Figure 3I). The MDSCs from tumor-bearing mice were sorted and pretreated with DOX before conducting the co-culture system to verify whether DOX affects the VM formation of B16-F10 cells by acting on the MDSCs (Figure 3J). Surprisingly, we found that DOX inhibited VM formation and VE-cadherin expression in B16-F10 cells (Figures 3K–3M).

### DOX inhibits MDSCs and enhances the anti-tumor effect of PD-1 inhibitor in B16-F10 mouse tumor models

**Figure 4:**
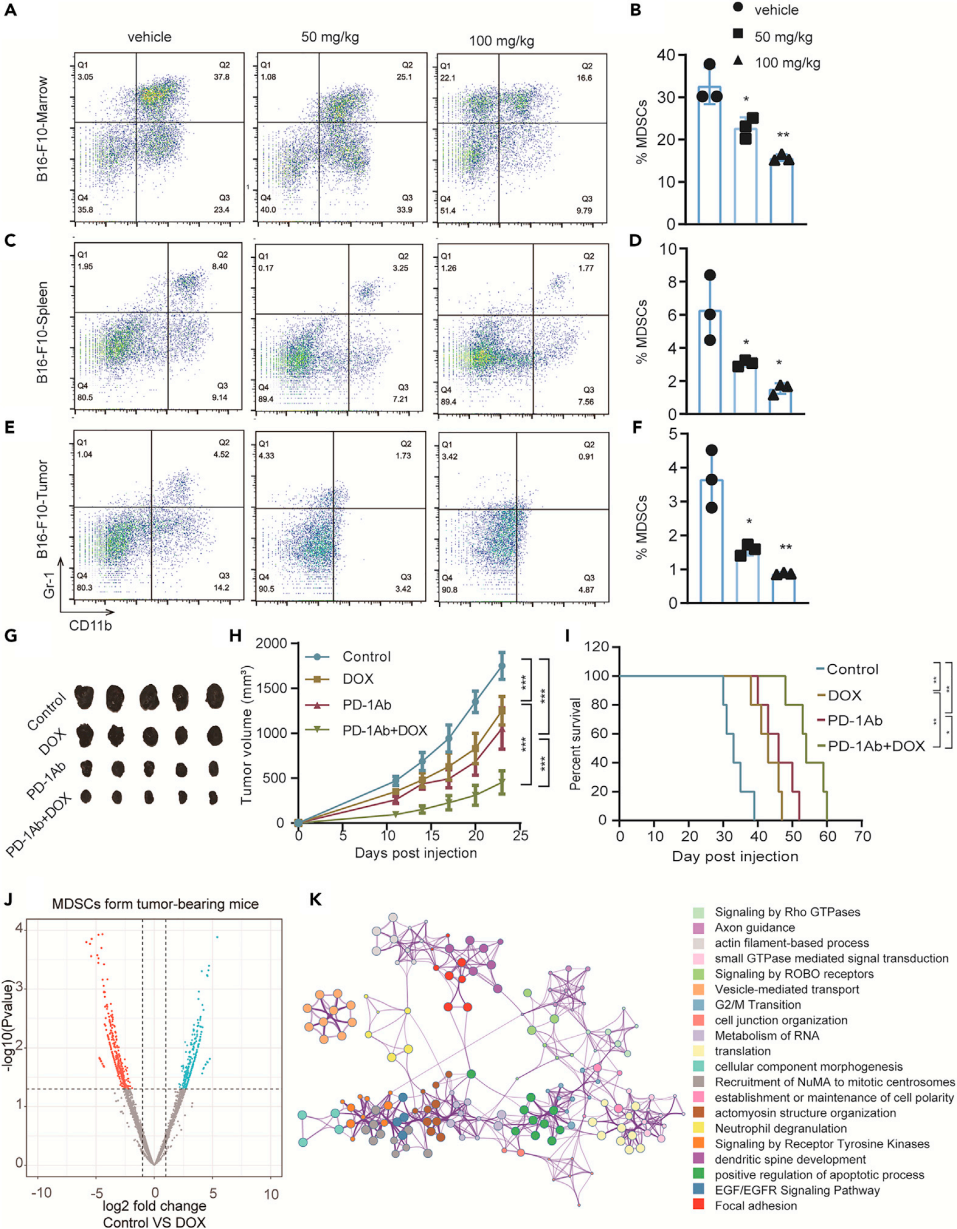
Doxycycline reduces the number of MDSCs and synergistically enhances the anti-tumor activity of PD1 in B16-F10 cell tumor-bearing model (A, C, and E) Dot plots of the proportion of MDSCs (CD11b^+^Gr-1^+^) in bone marrows, spleens, and tumor cells from control and DOX-treated B16-F10 tumor-bearing mice (n = 3). (B, D, and F) Unpaired Student’s t test was done for statistical analyses of ratio of MDSCs. All data are represented as mean ± SEM. ∗p < 0.05, ∗∗p < 0.01, ∗∗∗p < 0.001. (G) Tumor images of four groups to evaluate the anti-tumor effect of combination drug therapy of DOX and PD-1. (H) Tumor growth curves and statistical analysis of the four groups. (I) Survival curve of the four groups. Survival analysis was performed by using the Kaplan-Meier method with log rank test. ∗p < 0.05, ∗∗p < 0.01. (J) Volcano map of the up- and downregulated proteins of MDSCs. (K) Enrichment analysis of the differential proteins.

Flow cytometry was used to detect the proportion of MDSCs in the bone marrow, spleen, and tumor cells of the control and DOX-treated mice to investigate the dose-dependent effect of this drug on the immunosuppressive microenvironment. Reduced proportions of MDSCs were found in the bone marrow, spleen, and tumors (Figures 4A–4F). To further study which subsets of MDSCs DOX has an inhibitory effect on, we labeled the MDSCs by labeling CD11b, Ly6G, and Ly6C. We found that DOX inhibited PMN-MDSCs significantly, whereas it had no effect on M-MDSCs *in vivo* (Figure S2A–S2F), which is consistent with the results *in vitro*. A C57BL6 mouse model of subcutaneous transplantation tumor with B16-F10 cell line was established to detect the anti-tumor effect of combined DOX and PD-1 inhibitor. The combination therapy showed better anti-tumor effect than DOX or PD-1 inhibitor alone (Figures 4G–4I). The MDSCs from the bone marrow of B16-F10 tumor-bearing mice were then sorted, and some were treated with DOX *in vitro* for proteomic analysis to identify the pathways involved in the anti-tumor effects of DOX. A total of 837 differential proteins were detected. Enrichment analysis showed that many pathways involving signal transduction, cytoskeletal remodeling, and cell cycle were enriched (Figures 4J and 4K, Table S2).

### DOX inhibits MDSCs and enhances the anti-tumor effect of PD-1 inhibitor in 4T1 mouse breast tumor models

**Figure 5:**
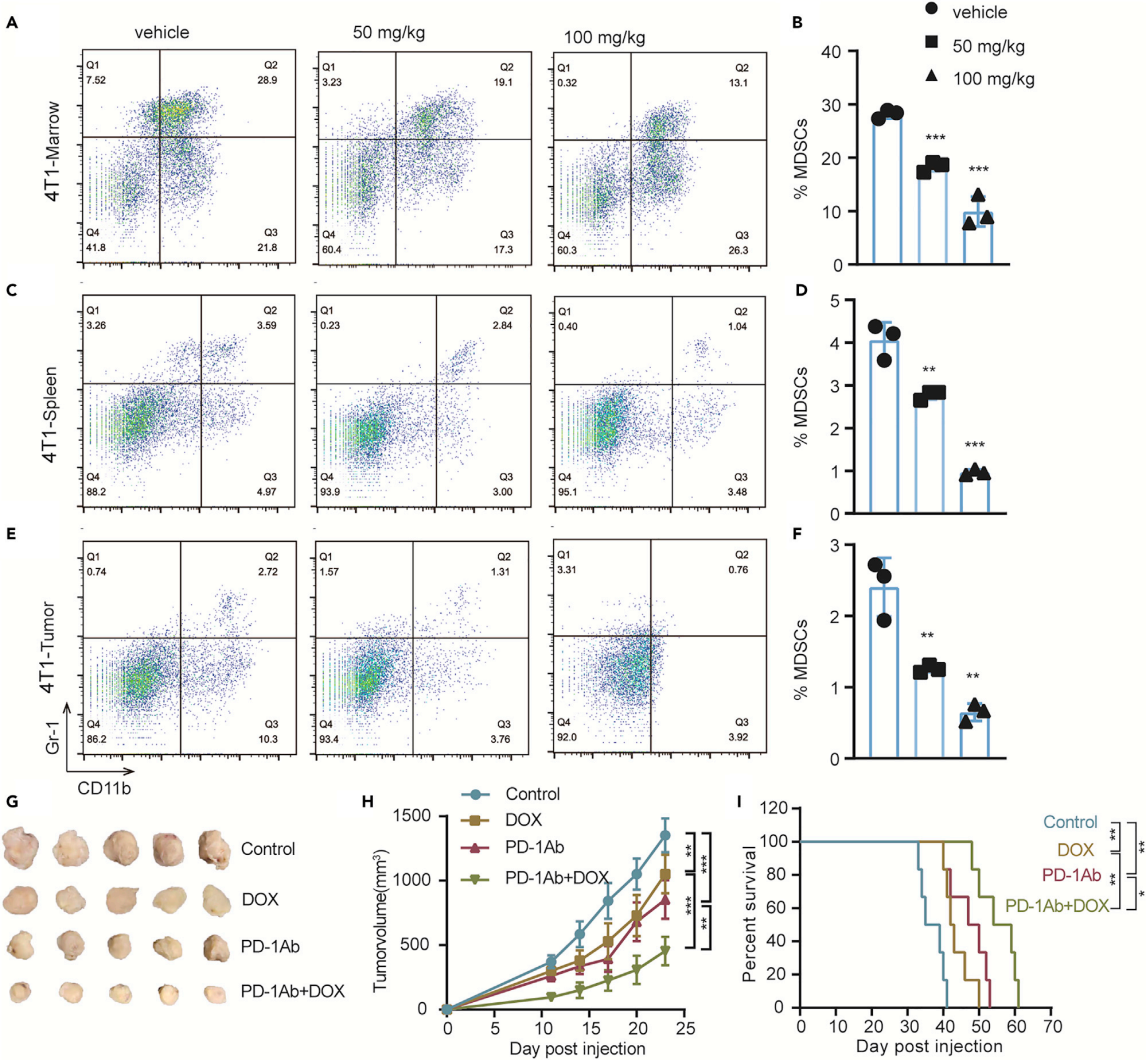
Doxycycline reduces the number of MDSCs and synergistically enhances the anti-tumor activity of PD1 in 4T1 tumor-bearing model (A, C, and E) Dot plots of the proportion of MDSCs (CD11b^+^Gr-1^+^) in bone marrows, spleens, and tumor cells from control and DOX-treated 4T1 tumor-bearing mice (n = 3). (B, D, and F) Unpaired Student’s t test was done for statistical analyses of ratio of MDSCs in 4T1 tumor-bearing model. All data are represented as mean ± SEM. ∗∗p < 0.01, ∗∗∗p < 0.001. (G) Tumor images of four groups to evaluate the anti-tumor effect of combination drug therapy of DOX and PD-1 in 4T1 tumor-bearing model. (H) Tumor growth curves and statistical analysis of the four groups. The data are represented as mean ± SEM. ∗∗p < 0.01, ∗∗∗p < 0.001(Student t-test). (I) Survival curve of the four groups. Survival analysis was performed by using the Kaplan-Meier method with log rank test. ∗p < 0.05, ∗∗p < 0.01, ∗∗∗p < 0.001.

A triple-negative breast cancer model (4T1-BALB/C) of subcutaneous transplantation tumor with 4T1 cell line was established to determine whether DOX can inhibit MDSCs in different tumors. The proportions of MDSCs in the bone marrows, spleens, and tumors of mice were then compared between the control and DOX-treated groups. Reduced proportions of MDSCs in bone marrows, spleens, and tumors were found after DOX administration (Figures 5A–5F). To further study which subsets of MDSCs that DOX has an inhibitory effect on, we labeled the MDSCs by labeling CD11b, Ly6G, and Ly6C. We found that DOX can inhibit PMN-MDSCs significantly, whereas it has no effect on M-MDSCs *in vivo* (Figure S3A–S3F). Tumor volume (Figures 5G and 5H) and mouse survival (Figure 5I) were also measured after the mice were treated with DOX and the combination of DOX and PD-1 inhibitor. The results showed that the combination treatment of DOX and PD-1 inhibitor significantly inhibited the tumor growth and prolonged the survival time.

## Discussion

Myeloid cells undergo abnormal differentiation in pathological conditions, especially during tumorigenesis, to form MDSCs that are highly concentrated in tumors and peripheral lymphoid organs (Ueha et al., 2011). MDSCs can be used as a marker of tumor immunosuppression microenvironment (Chun et al., 2015; Wang et al., 2016). These cells inhibit lymphocytes and cause immune escape by producing Arg-1, iNOS, and ROS. In addition to T cell suppression, MDSCs can also interact with tumor cells and directly promote the malignant progression of tumors. The tumor regulation of MDSCs is bidirectional; the activated MDSCs secrete chemokines, cytokines, and enzymes that contribute to tumor cell invasion, proliferation, survival, adhesion, and chemoattraction, thereby resulting in a cross talk that can affect tumor progression, invasion, and metastasis (Talmadge and Gabrilovich, 2013).

Tumor growth is closely related to blood supply and immunosuppression. MDSCs can stimulate the proliferation of endothelial cells (Talmadge and Gabrilovich, 2013; Yang et al., 2004), but many aggressive malignant tumors do not have endothelial cell-dependent blood vessels in the early stage (Sun et al., 2007a; Zhang et al., 2006). Instead, the tumor cells establish early microcirculation through VM formation. This study proved that MDSCs can promote VM formation and explained the early immunosuppression of aggressive malignant tumors. The endothelial-free and tube-like structures formed by tumor cells provide a blood channel for nutrient and oxygen supply that facilitates malignant progression (Nozawa et al., 2006).

The presence of VM is closely related to the occurrence, development, metastasis, and poor prognosis of tumor (Frenkel et al., 2008; Maniotis et al., 1999; Sun et al., 2012). This work attempted to establish the connection between MDSCs and VM by designing a co-culture system. B16-F10 cells exhibited stronger abilities of proliferation and VM formation in the presence of MDSCs than that in the absence of MDSCs. Therefore, an administration strategy of inhibiting the proliferation or function of MDSCs to weaken tumor cell VM formation can be designed.

DOX, a kind of semi-synthetic tetracycline drugs, can be used clinically to prevent and treat infections, periodontitis, and acne. DOX has high anti-VM activity and low cytotoxicity in melanoma and breast carcinoma (Liu et al., 2015; Sun et al., 2009). This drug also exhibits a suppressing effect through various pathways, such as MMPs (Sun et al., 2007b), FAK (Sun et al., 2009), and PAR1/NF-kB/miRNA-17 (Meng et al., 2014; Qin et al., 2015; Zhong et al., 2017) to target tumor cells. However, its role in tumor immunotherapy has not been reported. Here we found that DOX inhibited the proliferation and function of MDSCs.

In addition to DOX, previous study has found that 5-FU has an inhibitory effect on MDSCs (Pilot et al., 2020; Vincent et al., 2010). In the present study, we found that the inhibitory effect of DOX was comparable with that of 5-FU. For the subsets of MDSCs, DOX can selectively inhibit the PMN-MDSCs, whereas 5-FU is not selective for different subsets. For both comparisons, DOX has lower cytotoxicity than chemotherapeutics. We believe that DOX has the potential as an inhibitor of MDSCs to achieve antitumor effects and prevent the VM formation of tumor cells. Even as an old drug, DOX has presented a new application and became an investigational new drug (IND number: 2016L03513) in China as an antitumor drug that has entered clinical trials with the joint application of tumor immunotherapy.

As emerging antitumor drugs, PD-1 inhibitors have fewer overall side effects than traditional chemoradiotherapy but have the disadvantage of low efficiency. Once the PD-1 inhibitors take effect, some patients could achieve good clinical cure, that is, a 5- to 10-year survival time without recurrence and progression. Given that MDSCs have partly limited immune checkpoint inhibitors, the combination therapies can increase the response rates of PD-1/PD-L1 inhibitors (Gao et al., 2019). In this work, combination therapy of PD-1 inhibitor and DOX synergistically enhance antitumor effects. This study provides potential options for clinical settings to improve the antitumor effect of immunotherapy for cancer treatment.

### Limitations of the study

The molecular mechanism by which DOX inhibits MDSCs is not fully discovered and is currently being investigated. Although DOX promoted the apoptosis of MDSCs in this study, the inhibition efficiency is modest. Other pathways leading to MDSC death may also be involved. MDSCs may also transdifferentiate into other immune cells after DOX stimulation, such as converting into APC cells and then activating immunity (Ko et al., 2009). In addition, there are sex differences in the accumulation of MDSCs in tumor models, with females having higher levels of PMN-MDSCs (Bayik et al., 2020; Gabrilovich, 2020). As our studies used only female mice, sex differences should be considered for future studies as males may have a different response than what has been observed here, and this is a limitation of our experimental model.

## STAR★Methods

### Key resources table

**Table:** 

REAGENT or RESOURCE	SOURCE	IDENTIFIER
**Antibodies**		

anti-mouse CD16/32 mAb	BD Biosciences	Cat#553142; RRID: AB_394656
CD45	eBioscience	Cat#17-0459-42; RRID: AB_10667894
CD11b	eBioscience	Cat#12-0112-82; RRID: AB_2734869
Gr-1	eBioscience	Cat#11-5931-82; RRID: AB_465314
Ly-6G	eBioscience	Cat#17-9668-82; RRID: AB_2573307
Ly-6C	eBioscience	Cat#48-5932-82; RRID: AB_10805519
CD3	eBioscience	Cat#11-0031-82; RRID: AB_464882
CD8	eBioscience	Cat#12-0081-82; RRID:AB_465530
CD11b	Invitrogen	Cat#PA5-90724; RRID:AB_2806205
Gr-1	eBioscience	Cat#14-5931-86; RRID: AB_467732
iNOs	Affinity Bioreagents	Cat#AF0199; RRID: AB_2833391
Arg-1	Affinity Bioreagents	Cat#DF6657; RRID: AB_2838619
Goat anti-rabbit IgG (H+L) HRP	Affinity Bioreagents	Cat#S0001; RRID:AB_2839429
Goat anti-mouse IgG (H+L) HRP	Affinity Bioreagents	Cat#S0002; RRID:AB_2839430
beta Actin	Affinity Bioreagents	Cat#AF7018; RRID: AB_2839420
TRITC-conjugated secondary antibody	Invitrogen	Cat#T-2769; RRID: AB_2556777
FITC-conjugated secondary antibody	Invitrogen	Cat#A18866; RRID: AB_2535643
VE-cadherin	Affinity Bioreagents	Cat#AF6265; RRID: AB_2835123
Anti-mouse PD-1	Bio X Cel	Cat#BP0146; RRID:AB_10949053
Rat IgG2a	Bio X Cel	Cat#BP0089; RRID:AB_1107769

**Chemicals, peptides, and recombinant proteins**		

Collagenase I	Sigma-Aldrich	Cat#SCR103; CAS:9001-12-1
DNase I	Sigma-Aldrich	Cat#11284932001; CAS:9003-98-9
Bovine serum albumin (BSA)	Solarbio	Cat#A8010; CAS:9048-46-8
Recombinant GM-CSF	Novoprotein	Cat#CJ46; Accession:P01587
IL6	Novoprotein	Cat#CG39; Accession:P08505
2-mercaptoethanol	Biotech	Cat#M131-100ML; CAS:60-24-2
Doxycycline (DOX)	TargetMol	Cat#T1687-25 mg; CAS:564-25-0
Cocktail	Sigma-Aldrich	Cat#P2714
Roswell Park Memorial Institute (RPMI) 1640	HyClone	Cat#SH30809.01B
Fetal bovine serum (FBS)	HyClone	Cat#SH30084.03
Matrigel	BD Biosciences	Cat#354262
DiI cell-labeling solution	Invitrogen	Cat#V-22885
4% paraformaldehyde	SparkJade	Cat#EE0001
Crystal violet staining solution	KeyGEN	Cat#KGA229; CAS#: 603-48-5
Coomassie brilliant blue G-250	Solarbio	Cat#C8420; CAS:6104-58-1
Trypsin Protease	Thermo Scientific	Cat#90057
carboxyfluorescein succinimidyl ester (CFSE)	Sigma-Aldrich	Cat#21888; CAS:150347-59-4
5-Fluorouracil (5-FU)	Solarbio	Cat#F8301; CAS:51-21-8

**Critical commercial assays**		

Enhanced chemiluminescence detection kit	Vazyme	Cat#E411-05
Mycoplasma PCR Detection Kit	Beyotime	Cat#C0301S
Annexin V/PI apoptosis detection kit	KeyGEN	Cat#KGA101
ARG1 activity assay kit	Abcam	Cat#ab180877
H2DCFDA	Invitrogen	Cat#D399
Griess reagent system	Promega	Cat#G2930

**Deposited data**		

Mass spectrometry proteomics data submitted to ProteomeXchange	This study	ProteomeXchange: PXD027863

**Experimental models: Cell lines**		

Mouse:B16-F10	KeyGen Biotech	Cat#KG078
Mouse:4T1	KeyGen Biotech	Cat#KG338

**Experimental models: Organisms/strains**		

C57BL/6	Animal Center of the Academy of Military Medical Sciences	NA
BALB/C	Animal Center of the Academy of Military Medical Sciences	NA

**Software and algorithms**		

Maxquant v2.0	(Cox and Mann, 2008)	https://www.maxquant.org/
Metascape	(Zhou et al., 2019)	http://metascape.org
GraphPad Prism7	GraphPad	https://www.graphpad.com/

### Resource availability

#### Lead contact

Further information and requests for resources and reagents should be directed to and will be fulfilled by the Lead Contact, Tao Sun (tao.sun@nankai.edu.cn).

#### Material availability

This study did not generate new unique reagents.

### Experimental models and subjects

#### Primary cell cultures

After the mice were sacrificed, bone marrow cells collected from their femur and tibia were flushed with PBS by using a syringe. Spleen samples were processed through mechanical dissociation, and tumor tissues were transformed into single-cell suspensions by enzymatically dissociating the tissues for 1 h with 1 mg/mL type I collagenase (Sigma Aldrich, Saint Louis, U.S.A) in the presence of 50 units/mL DNase (Sigma Aldrich, Saint Louis, U.S.A). The cells were lysed with red blood cell lysis buffer, and the single-cell suspensions were prepared by using 100 μm filters.

#### Cell lines

Cell lines B16-F10 and 4T1 were purchased from KeyGen Biotech (Nanjing, China), verified by STR profiling and tested by mycoplasma detection (Beyotime, Beijing, China). The cells were then cultured with RPMI 1640 (HyClone, Logan, U.S.A) with 10% fetal bovine serum (HyClone, Logan, U.S.A) without antibiotics at 37°C in a humidified atmosphere containing 5% CO_2_.

#### Animals

Female C57BL/6 and BALB/C mice (6 weeks old) were purchased from the Animal Center of the Academy of Military Medical Sciences (Beijing, China), maintained in a temperature-controlled room with a 12 h/12 h light/dark schedule, and fed ad libitum. All animal experiments conformed to the guidelines of the Animal Ethics Committee of Tianjin International Joint Academy of Biotechnology and Medicine.

### Method details

#### Flow cytometry

The samples were washed with 1% bovine serum albumin (BSA) (Solarbio, Beijing, China) in PBS, blocked by non-specific staining with Fc block (anti-mouse CD16/32 mAb; BD Biosciences, Franklin Lakes, U.S.A), and stained with fluorescence-conjugated antibodies against surface markers CD45 (clone 30-F11), CD11b (clone M1/70), Gr-1 (clone 11-5931-81), Ly6G (clone 1A8-Ly6g), Ly6C(clone HK1.4), CD3 (clone 145-2C11), and CD8 (clone 53-6.7) (eBioscience, California, U.S.A) and detected by flow cytometry (BD LSRFortessa, Franklin Lakes, U.S.A).

#### Sorting of MDSCs

For the general population of MDSCs, the single-cell suspensions of bone marrow were stained with fluorescence-conjugated antibodies against the surface markers CD11b and Gr-1 for 30 min at 4°C. For the subsets of MDSCs, The single-cell suspensions of bone marrow were stained with fluorescence-conjugated antibodies against the surface markers CD11b，Ly6G and Ly6C for 30 min at 4°C. The MDSCs were sorted through flow cytometry (BD AriaIII, Franklin Lakes, U.S.A) and cultured in RPMI 1640 with 10% fetal bovine serum, 20 ng/mL recombinant GM-CSF (recombinant CJ46, Novoprotein, Shanghai, China), 20 ng/mL IL6 (CG39, Novoprotein, Shanghai, China), and 50 μM 2-mercaptoethanol (Biotech, Hangzhou, China).

#### Western blot analysis

The MDSCs were sorted from the bone marrows of the B16-F10 tumor-bearing mice and cultured in MDSC culture medium. The cells were divided into three groups, namely, control, DOX-low (2.5 μM), and DOX-high (5 μM) groups. After the cells were treated with DOX for 48 h, the expression of iNOS and Arg-1 in MDSCs was determined by Western blot analysis. After 24 h, the cells were treated with drugs for 24 h or 48 h, washed with PBS, and lysed in ice-cold lysis buffer with protease inhibitor cocktail (Sigma Aldrich, Saint Louis, U.S.A) for 30 min. The lysates were separated through SDS-PAGE and then transferred to PVDF membranes (Millipore, Massachusetts, USA). These membranes were then blocked and incubated first with primary antibodies Arg-1 (1:1000, Affinity Bioreagents, Colorado, U.S.A) and iNOS (1:1000, Affinity Bioreagents, Colorado, U.S.A) and subsequently with secondary antibodies (1:5000, Affinity Bioreagents, Colorado, U.S.A). The blots were visualized with an enhanced chemiluminescence detection kit (Vazyme, Nanjing, China).

#### *In vivo* experiment

In brief, 4 × 10^5^ cells were resuspended in 0.1 mL of PBS and subcutaneously injected into the right lateral flank of C57BL/6 mice to establish the B16-F10 tumor model. Another batch of resuspended 4 × 10^5^ 4T1 cells in 0.1 mL of PBS were injected into the fourth pair of the mammary fat pad of BALB/C mice to establish the 4T1 tumor model. When the tumor sizes reached 120-180 mm^3^, the animals were randomly assigned into three groups (n = 6), namely, control, DOX-low, and DOX-high groups that were intragastrically administered with the vehicle (normal saline, Solarbio, Beijing, China) or 50 or 100 mg/kg/day DOX , respectively, for 14 days.

#### VM formation assay

The 48-well plates were coated with Matrigel (BD Biosciences, Franklin Lakes, USA) and polymerized at 37°C for 2 h. After being co-cultured with MDSCs for 24 h, approximately 1 × 10^5^ B16-F10 cells were obtained and then cultured in the Matrigel-coated plates for 12 h. Random fields were observed with a fluorescence microscope (Nikon, Tokyo, Japan), and the number of tubes was counted. The experiment was independently repeated three times.

#### Immunofluorescence analysis

The fixed cells sorted from the marrow were blocked with 1% BSA for 30 min and incubated with anti-Gr-1(1:200, eBioscience, California, U.S.A) and anti-CD11b(1:200, eBioscience, U.S.A) antibody for 1 h to detect the level of MDSCs in the marrow. For double labeling, fluorescent labeling was performed with a TRITC-conjugated secondary antibody and a FITC-conjugated secondary antibody to detect MDSCs. The B16-F10 cells stably expressing the enhanced expressing green fluorescent protein were co-cultured with MDSCs and labeled by the fluorescent probe DiI (Molecular Probes, Invitrogen, California, U.S.A). Living cell tracing was conducted for the co-cultured cells for 10 h. The co-cultured cells were then fixed with 4% paraformaldehyde for 5 min at room temperature and blocked with 1% BSA for 30 min to detect VE-cadherin expression in B16-F10 cells with or without MDSCs. Primary antibody anti-VE-cadherin (1:200, Affinity Bioreagents, Colorado, U.S.A) was used to label VE-cadherin. The images were observed with a confocal microscope (Nikon, Tokyo, Japan).

#### Transwell assay

The cells subjected to different treatments were added in top-chamber inserts coated with Matrigel (BD Biosciences, Franklin Lakes, USA). The lower chamber was incorporated with 500 μL of the medium supplemented with 10% FBS and served as a chemotactic agent. After being cultured at 37°C for 24 h, the cells invading the lower chamber were fixed in 4% paraformaldehyde (pre-cooled at 4°C), stained with crystal violet staining solution (KeyGEN, Nanjing, China), and counted under an upright microscope (five fields per chamber).

#### Proteomics data analysis

The MDSCs from the bone marrows of B16-F10 tumor-bearing mice and the naive mice were sorted and cultured *in vitro* to determine the effect of DOX (5 μM). Label-free quantitative proteomics was used to determine differential expression. 30 μg protein extracted form MDSCs was loaded onto the SDS-PAGE and run into the gel at approximately 2 cm. The gel stained by Coomassie blue was cut, destained, subjected to in-gel digestion using trypsin (Thermo Scientific, Waltham, U.S.A), and analyzed by LC−MS/MS using fragmentation by higher-energy collisional dissociation (Triple-quad Ion-trap and Orbitrap fusion, Thermo Scientific, Waltham, U.S.A). Raw data were examined using free academic software Maxquant to quantify the expression(Cox and Mann, 2008). A fold change of more than 1 was defined as significantly different. Pathway and process enrichment analyses were conducted by using Metascape (http://metascape.org) (Zhou et al., 2019). The mass spectrometry proteomics data have been deposited in ProteomeXchange Consortium via the PRIDE partner repository with the dataset identifier PXD027863.

#### Cell proliferation assay

For the detection of co-cultured cells，the MDSCs were sorted from the bone marrows of the B16-F10 tumor-bearing mice and cultured in MDSC medium. The B16-F10 cells were stained with 5 μM carboxyfluorescein succinimidyl ester (CFSE; Sigma Aldrich, Saint Louis, U.S.A) and co-cultured with MDSCs for 24 h. CFSE dilution was determined by flow cytometry. All results were expressed as mean ± SEM. For the detection of PMN-MDSCs, The PMN-MDSCs were sorted from the bone marrows of the B16-F10 tumor-bearing mice and were stained with 5 μM CFSE.

#### Cell apoptosis assay

The MDSCs and CD8 T lymphocytes from the bone marrows and spleens of B16-F10 tumor-bearing mice were sorted and cultured in MDSC and T-lymphocyte media, respectively, to determine the effect of DOX on MDSCs and CD8 T lymphocytes. The MDSCs and CD8 T lymphocytes were then divided into three groups, namely, control, DOX-low (2.5 μM), and DOX-high (5 μM). After 48 h, the cells were stained with an Annexin V/PI apoptosis detection kit (KeyGen Biotech, Nanjing, China), incubated in the dark for 30 min, and analyzed by flow cytometry. All results were expressed as mean ± SD.

#### Arg-1, reactive oxygen species (ROS) and NO detection

The MDSCs from the bone marrows of naive and B16-F10 tumor-bearing mice were sorted, cultured in MDSC culture medium, and divided into three groups. ARG1 activity, ROS and NO were detected by using Arg-1 activity assay kit (Abcam, Cambridge, UK), H2DCFDA (Invitrogen, California, U.S.A), and Griess reagent system (Promega, Madison, U.S.), respectively, in accordance with the manufacturers' instructions.

#### Treatment of DOX combined with PD-1 inhibitor *in vivo*

The B16-F10 and 4T1 tumor models were established by using the methods described above. When the tumor volumes of B16-F10 and 4T1 tumor-bearing mice reached 120–180 mm^3^, the mice were randomly grouped as follows (n = 6): control, DOX, anti-PD-1 and DOX+anti-PD-1. The control group was treated with normal saline and the DOX groups were intraperitoneally administered with either 100 or 200 mg/kg DOX daily for 14 days. Anti-PD-1 antibody (clone RMP1-14, Bio X Cell, West Lebanon, U.S.A) or isotype control antibody (clone 2A3, rat IgG2a, Bio X Cell, West Lebanon, U.S.A) was intraperitoneally administered on days 11, 14, 17, 20, and 23 (200 μg/injection). Tumor volume was measured every 3 days and calculated as length × width^2^/2.

### Quantification and statistical analysis

All statistical analyses were performed with GraphPad Prism7 software for Windows. Statistically significant differences were calculated by using Student's t-test. Survival analysis was performed by using the Kaplan–Meier method with log-rank test, and p < 0.05 was considered statistically significant.

## Data Availability

• The mass spectrometry proteomics data were deposited to ProteomeXchange: PXD027863. • This paper does not report original code. • Any additional information required to reanalyze the reported data is available from the lead contact upon request.

## Supplemental information

Document S1. Figures S1–S3

Table S1. Differentially expressed proteins in the MDSCs of tumor-bearing mice compared with naive mice, related to Figure 1

Table S2. Differentially expressed proteins of DOX-treated MDSCs compared with vehicle-treated mice, related to Figure 4
