# Targeting myeloid-derived suppressor cells to attenuate vasculogenic mimicry and synergistically enhance the anti-tumor effect of PD-1 inhibitor

**DOI:** 10.1016/j.isci.2022.105281

**Published:** 2022-10-09

**Authors:** Yinan Li, Kailiang Qiao, Xiaoyun Zhang, Haoyang Liu, Heng Zhang, Zhiyang Li, Yanrong Liu, Tao Sun

## Main text

(iScience *24*, 103392, December 17, 2021)

During the figure preparation for this article, the wrong first panel in Figure 2C was inadvertently presented. This has now been corrected online.

The authors regret this error and apologize for any confusion that it has caused.

**Figure:**
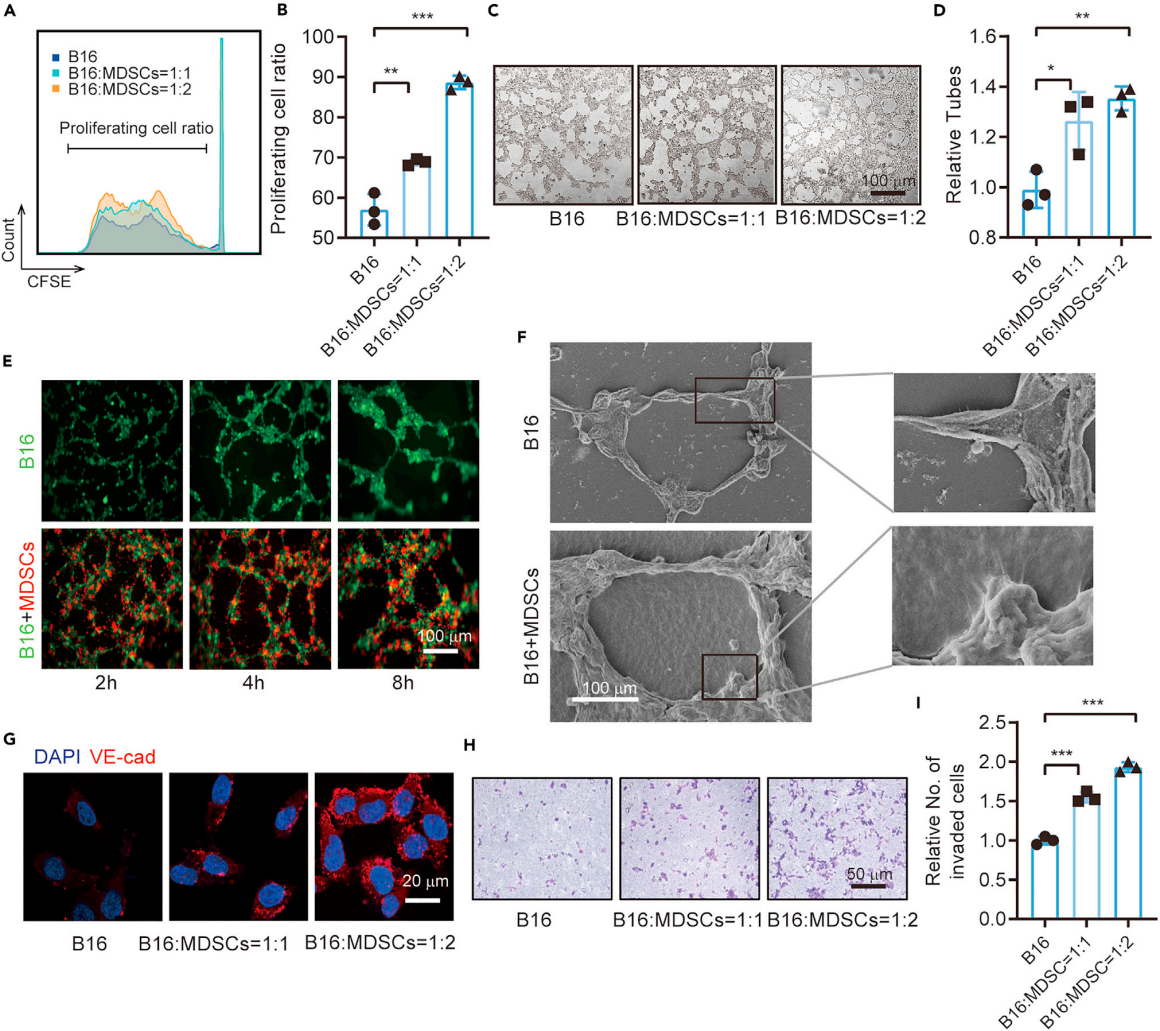
Figure 2. MDSCs promote proliferation and VM formation of B16-F10 cells *in vitro* (original)

**Figure:**
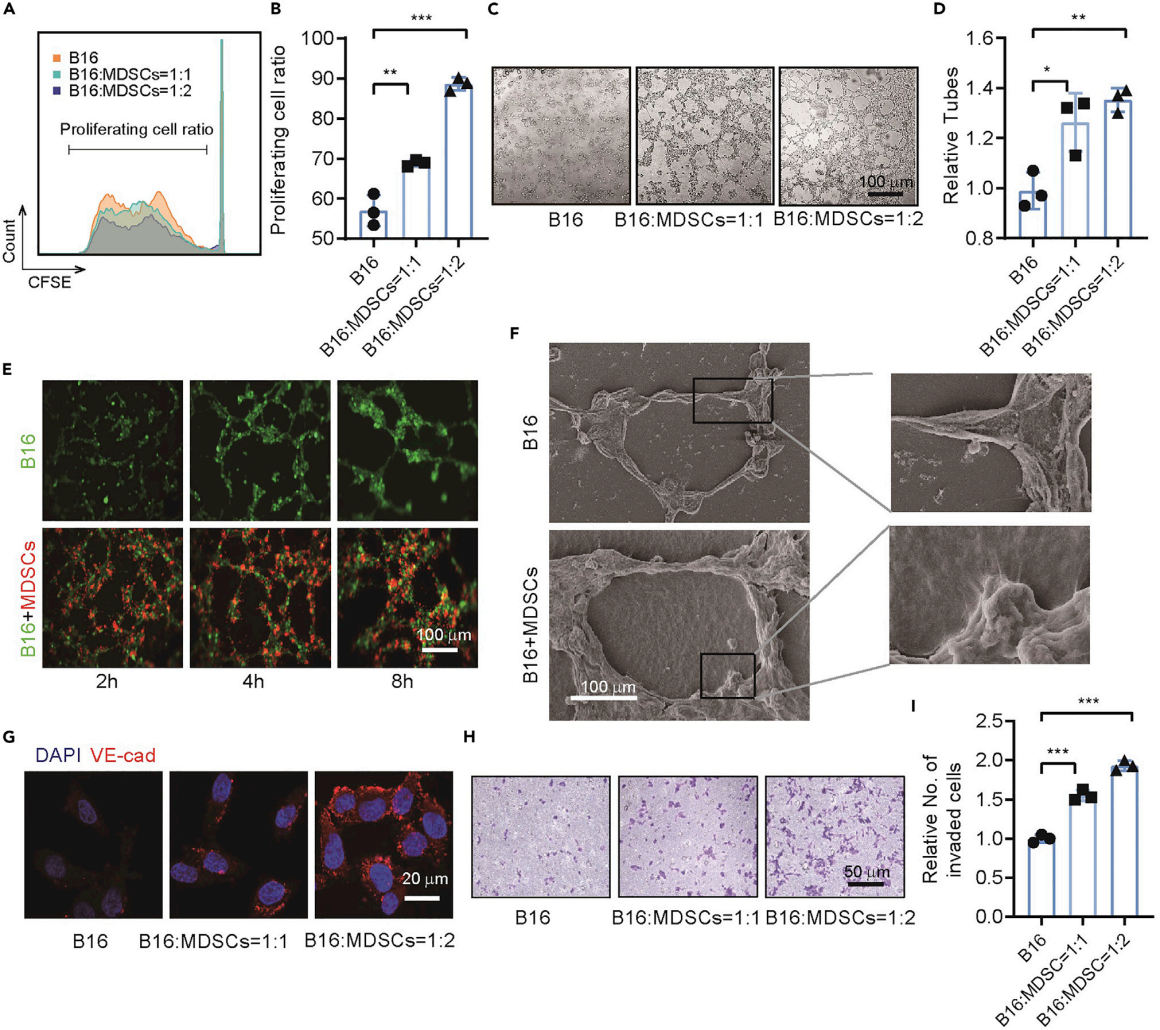
Figure 2. MDSCs promote proliferation and VM formation of B16-F10 cells *in vitro* (corrected)

