# Supplementary material for: Targeting myeloid-derived suppressor cells to attenuate vasculogenic mimicry and synergistically enhance the anti-tumor effect of PD-1 inhibitor
Source: iScience. 2021 Nov 1;24(12):103392. doi: 10.1016/j.isci.2021.103392 (PMC8605339; doi:10.1016/j.isci.2021.103392)
Supplement: Document S1. Figures S1–S3 [file mmc1.pdf]

iScience, Volume *24*

Supplemental information

Targeting myeloid-derived suppressor cells to attenuate vasculogenic mimicry and synergistically enhance the anti-tumor effect of PD-1 inhibitor

Yinan Li, Kailiang Qiao, Xiaoyun Zhang, Haoyang Liu, Heng Zhang, Zhiyang Li, Yanrong Liu, and Tao Sun

Figure S1. Apoptosis detection of MDSCs treated by DOX, Related to Figure 3.

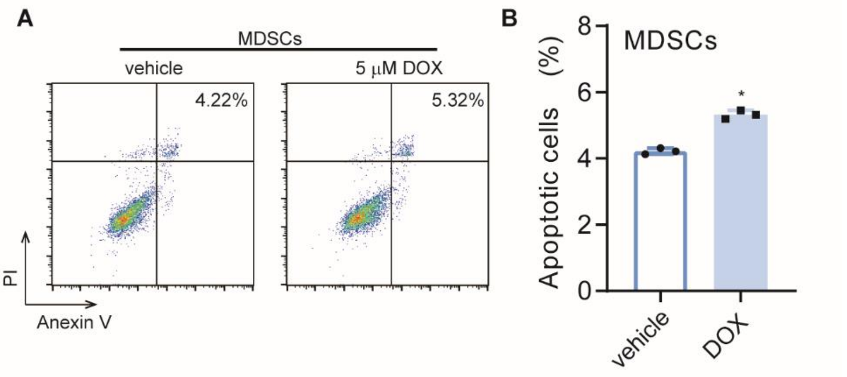

(A) Representative dot plots for apoptosis of MDSCs after DOX treatment. (B) Statistical analysis(Student t-test) of the apoptosis ratio of MDSCs. All statistical data are represented as mean ± SEM. *p < 0.05, **p < 0.01, ***p < 0.001.

Figure S2. Doxycycline reduces the number of PMN-MDSCs in B16-F10 cell tumor-bearing model, Related to Figure 4.

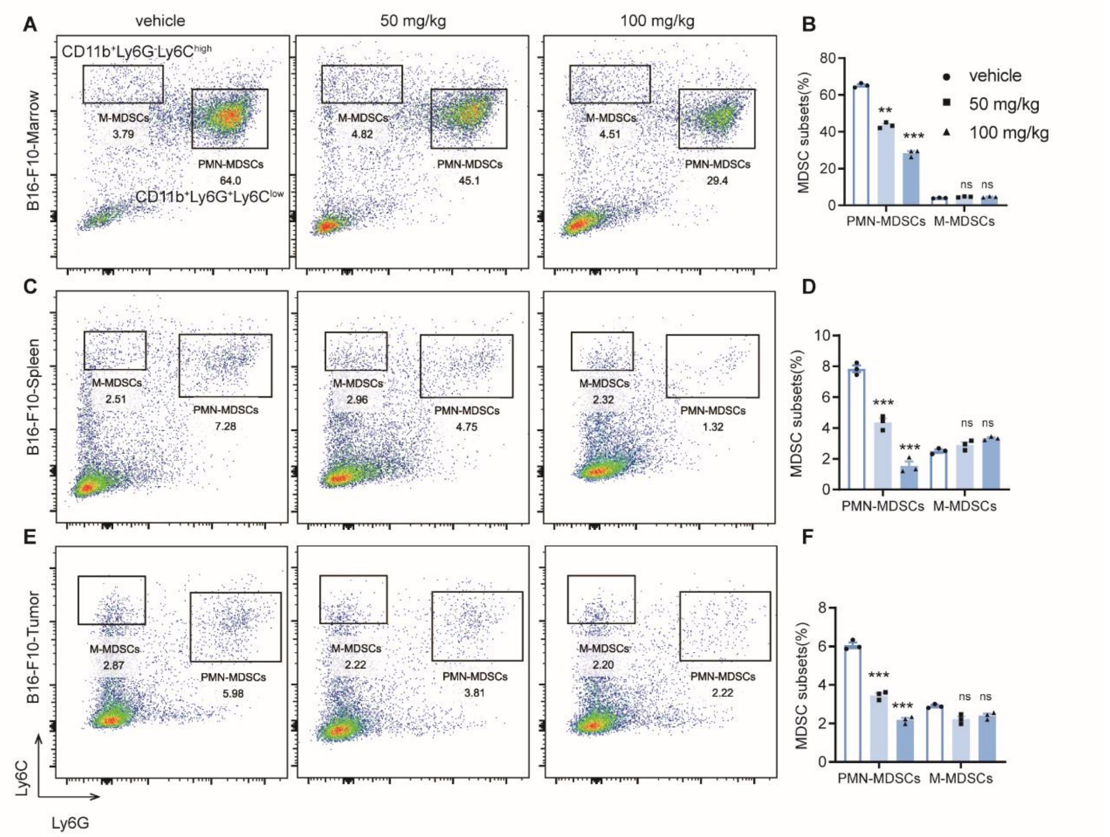

(A, C, and E) Dot plots of the proportion of PMN-MDSCs (CD11b^{+}Ly6G^{+}Ly6C^{low}) and M-MDSCs (CD11b^{+}Ly6G^{-}Ly6C^{high}) in bone marrows, spleens, and tumor cells from control and DOX-treated B16-F10 tumor-bearing mice (n = 3). (B, D, and F) Statistical analysis(Student t-test) of the ratio of MDSCs subsets. All statistical data are represented as mean ± SEM. *p < 0.05, **p < 0.01, ***p < 0.001.

Figure S3. Doxycycline reduces the number of PMN-MDSCs in 4T1 tumor-

bearing model, Related to Figure5.

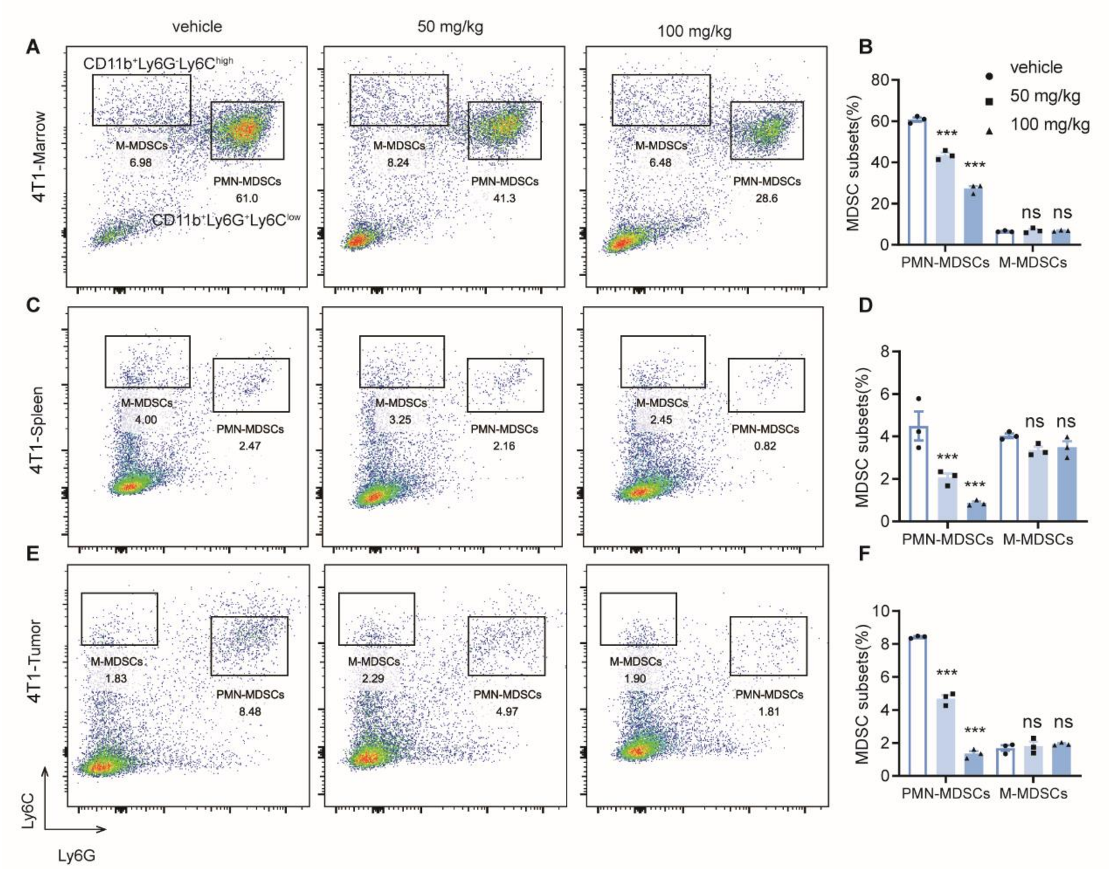

(A, C, and E) Dot plots of the proportion of PMN-MDSCs ($CD11b^{+}Ly6G^{+}Ly6C^{low}$) and M-MDSCs ($CD11b^{+}Ly6G^{-}Ly6C^{high}$) from control and DOX-treated 4T1 tumor-bearing mice (n = 3). (B, D, and F) Statistical analysis(Student t-test) of the ratio of MDSCs subsets in 4T1 tumor-bearing model. All statistical data are represented as mean ± SEM. *p < 0.05, **p < 0.01, ***p < 0.001.
